# *PTPRG* suppresses tumor growth and invasion via inhibition of Akt signaling in nasopharyngeal carcinoma

**DOI:** 10.18632/oncotarget.3876

**Published:** 2015-04-19

**Authors:** Arthur Kwok Leung Cheung, Joseph Chok Yan Ip, Adrian Chi Hang Chu, Yue Cheng, Merrin Man Long Leong, Josephine Mun Yee Ko, Wai Ho Shuen, Hong Lok Lung, Maria Li Lung

**Affiliations:** ^1^ Department of Clinical Oncology, University of Hong Kong, Hong Kong (SAR), People's Republic of China; ^2^ Centre for Cancer Research, University of Hong Kong, Hong Kong (SAR), People's Republic of China; ^3^ Centre for Nasopharyngeal Carcinoma Research, University of Hong Kong, Hong Kong (SAR), People's Republic of China; ^4^ Division of Medical Oncology, National Cancer Centre, Singapore

**Keywords:** PTPRG, Akt, EGFR, nasopharyngeal carcinoma, tumor suppressor

## Abstract

Protein Tyrosine Phosphatase, Receptor Type G (PTPRG) was identified as a candidate tumor suppressor gene in nasopharyngeal carcinoma (NPC). PTPRG induces significant *in vivo* tumor suppression in NPC. We identified EGFR as a PTPRG potential interacting partner and examined this interaction. Dephosphorylation of EGFR at EGFR-Y1068 and -Y1086 sites inactivated the PI3K/Akt signaling cascade and subsequent down-regulation of downstream pro-angiogenic and -invasive proteins (VEGF, IL6, and IL8) and suppressed tumor cell proliferation, angiogenesis, and invasion. The effect of Akt inhibition in NPC cells was further validated by Akt knockdown experiments in the PTPRG-down-regulated NPC cell lines. Our results suggested that inhibition of Akt in NPC cells induces tumor suppression at both the *in vitro* and *in vivo* levels, and also importantly, *in vivo* metastasis. In conclusion, we confirmed the vital role of PTPRG in inhibiting Akt signaling with the resultant suppression of *in vivo* tumorigenesis and metastasis.

## INTRODUCTION

The incidence of nasopharyngeal carcinoma (NPC) is highest in Southeast China and Asia. Epstein-Barr Virus (EBV) infection was found to closely associate with NPC development. EBV infection in the epithelial cells was reported to be crucial to induce host cell signaling changes during cancer progression. The EBV oncogenic latent gene, Latent Membrane Protein 1 (LMP1), can activate various signaling pathways, including the PI3K/Akt signaling pathway, and results in enhancing cell survival [1]. Furthermore, LMP1 up-regulates [2] and activates EGFR in NPC cells [3]. A positive correlation between the phosphorylated-EGFR and phosphorylated-Akt was detected in NPC patient tumors, suggesting a regulatory role of EGFR in Akt activation in NPC [4]. Inhibition of the EGFR by EGFR-specific tyrosine kinase inhibitors significantly suppressed NPC cell growth [5]. These previous studies suggested the importance of the EGFR/PI3K/Akt signaling in NPC.

Our previous NPC study identified a candidate tumor suppressor gene (TSG), *Protein Tyrosine Phosphatase, Receptor Type G* (*PTPRG*), which belongs to the tyrosine phosphatase (PTP) family. PTP family members are associated with cancer development and they function in an opposite manner to receptor tyrosine kinases (RTKs), which mainly participate in regulation of various crucial signaling pathways to control cell cycle progression, proliferation, invasion, and angiogenesis. The balances between the activities of PTP and RTK members are vital to maintain cell signaling homeostasis. Imbalance between the activities of PTPs and RTKs is critical for inducing malignant transformation of normal cells [6]. The classical PTPs are categorized into receptor, non-receptor, and also dual specificity phosphatase (DUSP) types [7]. Members in the PTP super family can be either oncogenic or tumor suppressive. The functional roles of PTPs are mainly determined by their subcellular localization and also their interacting partners.

Recently, various members in the receptor type PTP family were identified as tumor suppressors. Among them, PTEN is one of the best-studied members. PTEN can induce dephosphorylation of PI3K and inactivation of the PI3K/Akt signaling pathway activities. The PI3K/Akt pathway plays a major role in control of cell growth, proliferation, cell survival, angiogenesis, and invasion [7]. Mutations resulting in its deregulation are commonly observed in various cancers, including NPC.

In this current study, the tumor suppressive role of *PTPRG* was investigated. *PTPRG* point mutations have been identified in colon cancer patients [8]. Down-regulation and promoter hypermethylation are critical factors associated with *PTPRG* inactivation in cancer and have been observed in sporadic and Lynch syndrome colorectal cancer [9], ovarian, breast, and lung cancers [10], gastric cancer [11], chronic myeloid leukemia [12], T-cell lymphoma [13], and NPC [14]. Functional studies suggested that re-expression of PTPRG induced significant tumor suppressive effects in different cancers. Over-expression of *PTPRG* in breast cancer cells prolongs doubling times and colony sizes of breast cancer cells [15] and inhibits *in vivo* breast tumor formation through up-regulation of p21 and p27 by suppression of ERK1/2 [16]. PTPRG interacts and dephosphorylates the oncogenic fusion protein, BCR/ABL, to inactivate its downstream signaling molecules [12]. Our earlier NPC study also confirmed that re-expression of *PTPRG* suppressed *in vivo* tumor growth and induced cell cycle G_0_/G_1_ arrest by down-regulation of cyclin D1 protein levels and, thus, reduced phosphorylation of pRB [14]. However, the *PTPRG*-associated signaling changes remain unclear. Further studies are necessary to identify the role of this PTP in cancer development.

In this current study, we identified the potential PTPRG-associated signaling pathways and protein-interacting partners that impact its tumor and metastasis suppressive roles in NPC.

## RESULTS

### PTPRG reduces phosphorylation of the Akt signaling pathway cascade in NPCS

**Figure 1:**
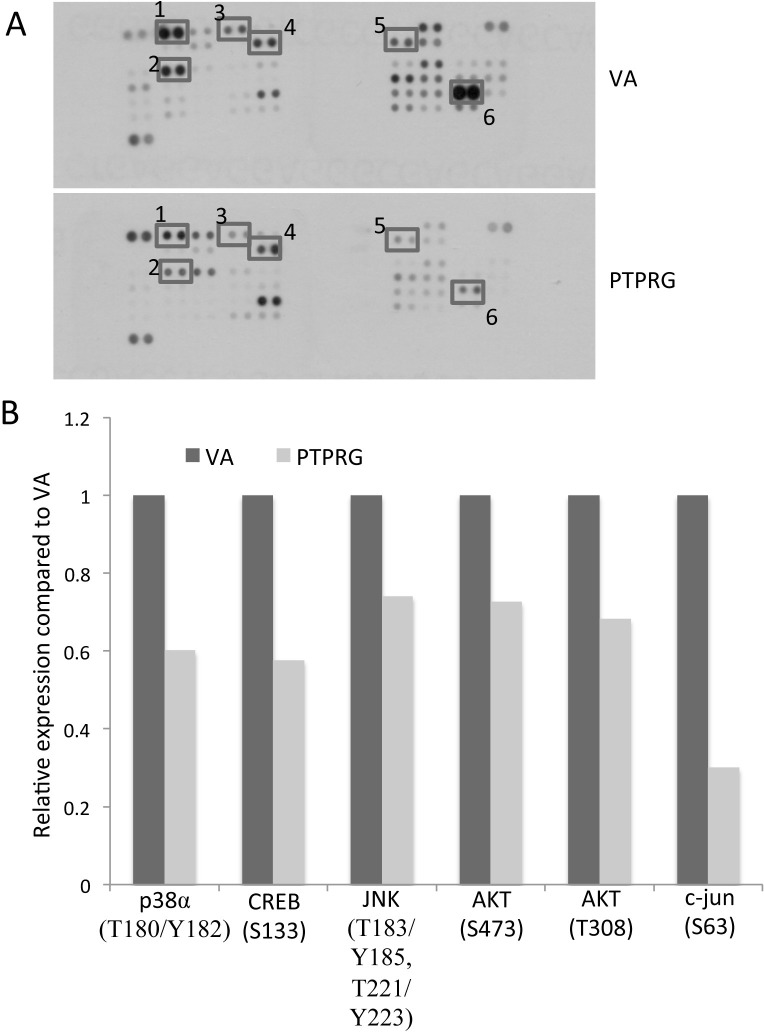
Protein kinase phosphorylation levels associated with PTPRG expression **A.** Changes of phosphorylation levels of Akt and its downstream molecules observed on the phosphorylation array of the vector-alone (VA) and PTPRG-expressing clones. Duplicate spots: 1) p38α (T180/Y182), 2) CREB (S133), 3) JNK (T183/Y185, T221/Y223), 4) Akt (S473), 5) Akt (T308), and 6) c-jun (S63). **B.** Relative phosphorylation levels of protein kinases differentially expressed in the PTPRG-expressing clones. Relative phosphorylation levels were calculated by comparing the intensity seen on the protein array of the PTPRG-expressing clones versus VA controls.

Previous studies showed PTPRG is involved in regulation of the ERK1/2 signaling pathway in breast cancer cells [16] and also cyclinD1/Rb in NPC [14]. In order to identify PTPRG-regulated targets in NPC, a human kinase phosphorylation-specific antibody array was utilized to examine the changes in phosphorylation levels of protein kinases after re-expressing PTPRG in NPC cells. A tumor-suppressive PTPRG-expressing clone and vector-alone (VA), which were previously established in the HONE1 NPC cell line [14] engineered with a tetracycline-regulated inducible (tet-off) system [17], were used for this antibody array study. Several changes were observed in the antibody array assay. In this current study, we focused on the changes of phosphorylation levels of Akt and its downstream molecules, as shown in Figure 1A and Supplementary Table 1. After hybridization of the protein lysates of VA and PTPRG-expressing clones to the antibody array, dramatic reduction of phosphorylation of p38α-T180/Y182, CREB-S133, JNK-T183/185, T221/223, Akt-S473 and -T308, and c-jun-S63, was detected (Figure 1A and 1B), providing evidence to support a role for PTPRG as an Akt signaling regulator in cancer.

### PTPRG co-immunoprecipitates with EGFR

**Figure 2:**
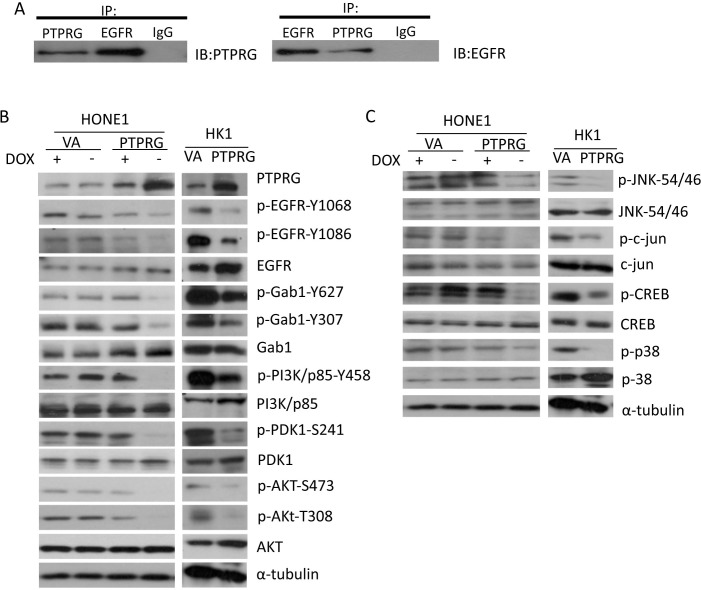
PTPRG interacts with EGFR and regulates the EGFR/PI3K/Akt signaling pathway **A.** CoIP assay was performed by utilizing the antibodies to PTPRG and EGFR. The IgG was used as a negative control. PTPRG showed interaction with the EGFR. **B.** Re-expression of PTPRG reduced phosphorylation of EGF/PI3K/AKT signaling pathway members (EGFR, Gab1, PI3K/p85, PDK1, and AKT) in both PTPRG-re-expressing HONE1 and HK1 NPC cell lines. The α-tubulin served as an internal loading control. **C.** PTPRG induced dephosphorylation of Akt downstream signaling molecules. PTPRG reduced the phosphorylation of AKT-downstream signaling members (JNK, c-jun, and CREB) and MAPK-downstream signaling member, p38, in both HONE1 and HK1. The α-tubulin served as an internal loading control.

PTPRG is classified as a membrane receptor [18]. The phosphorylation antibody array data indicated that re-expression of PTPRG reduced the phosphorylation not only at the tyrosine site of Akt, but also at both the Akt-S473 and -T308 phosphorylation sites (Figure 1A and 1B). As PTPRG is a tyrosine phosphatase, this suggested that PTPRG might not function alone to directly reduce Akt phosphorylation. Therefore, determining how PTPRG regulates the phosphorylation of the Akt signaling members was further investigated. We, thus, performed co-immunoprecipitation (CoIP) to identify PTPRG protein-interacting partner(s). CoIP results suggested that PTPRG directly interacts with a cell surface receptor, EGFR, which is one of the key cell surface receptors to activate the Akt signaling pathway (Figure 2A). Thus, EGFR is one of the potential PTPRG targets suppressing Akt signaling pathway phosphorylation.

### PTPRG reduces EGFR phosphorylation levels and regulates downstream PI3K/Akt and p38

EGFR phosphorylation was confirmed to induce Akt phosphorylation in NPC [4]. Taken together with the CoIP results, we investigated the role of PTPRG in regulating the EGFR/Akt signaling phosphorylation in NPC. In our previous NPC study, *PTPRG* was confirmed to be down-regulated in the NPC cell lines, including HONE1 and HK1 [14]. Therefore, these two NPC cell lines were used in these current studies. In the previously established HONE1-*PTPRG* inducible clone, in the absence of doxycycline (−Dox), PTPRG protein is expressed. In the presence of Dox (+Dox), PTPRG expression levels are down-regulated (Figure 2B). In the HK1 cell line, PTPRG was transiently expressed (Figure 2B).

This panel of PTPRG-expressing NPC cell lines was used to investigate the contribution of PTPRG in regulating the phosphorylation of EGFR and Akt signaling members. Phosphorylation levels of the two EGFR tyrosine sites, Y1068 and Y1086, were reduced in the PTPRG-expressing HONE1 and HK1 cells (Figure 2B). These two phosphorylation sites are responsible for EGFR-associated PI3K/Akt and MAPK signaling activation. Based on these results, phosphorylation levels of their expected downstream signaling targets were also investigated by Western blot (WB) analysis. Reduction of phosphorylation of EGFR downstream signaling molecules, including p-Gab1 (Y627 and Y307), PI3K/p-85 (Y458), p-PDK1 (S241), and Akt (S473 and T308) was observed in the PTPRG-expressing cells (Figure 2B). This suggested the ability of PTPRG to regulate Akt signaling through dephosphorylation of EGFR.

To further confirm the ability of PTPRG to regulate the Akt signaling, phosphorylation levels of Akt downstream targets, including p-JNK, p-c-jun, and p-CREB were investigated. Results suggested that their phosphorylation was greatly reduced when PTPRG was expressed (Figure 2C). Furthermore, one of the MAPK signaling members, p38, which also showed decreased phosphorylation levels in the PTPRG phosphorylation antibody array, showed a lower phosphorylation level after PTPRG expression (Figure 2C). This further confirmed the ability of PTPRG to regulate the phosphorylation of the EGFR to suppress the downstream signaling pathway.

### Akt inhibition in the PTPRG-down-regulated NPC cells induces *in vivo* tumor suppression

**Figure 3:**
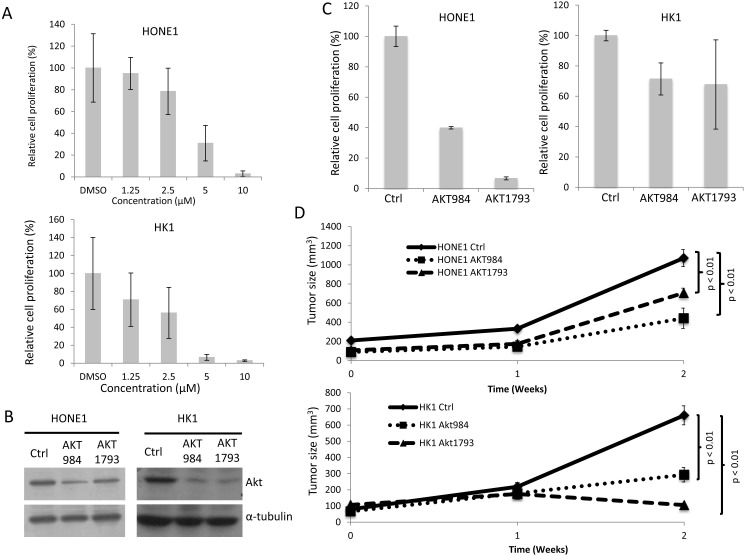
Targeting Akt inhibited *in vitro* cell proliferation and *in vivo* tumor formation **A.** *In vitro* cell proliferation assay of the HONE1 and HK1 treated with Akt inhibitor. Different concentrations of the Akt inhibitor (1.25 μM, 2.5 μM, 5 μM, and 10 M) and DMSO control were used for the treatment of the two NPC cell lines. The relative proliferation rate was compared to the DMSO control. Data are represented as the mean + SD. **B.** Akt protein levels in the HONE1 and HK1 cells after Akt knockdown. The scramble control (Ctrl) and two sets of Akt knockdown oligonucleotides, AKT984 and AKT1793, were used in the knockdown experiments. **C.** *In vitro* cell proliferation assay of HONE1 and HK1 after Akt knockdown. The relative proliferation rate was compared to the scramble control (Ctrl). Data are represented as mean + SD. **D.** *In vivo* tumorigenicity assay of HONE1 and HK1 after Akt knockdown. The curves represent an average tumor volume of all six injection sites. Data are represented as mean + SEM. The Ctrl formed large tumors and the Akt knockdown resulted in reduced tumor sizes.

Akt signaling is a key signaling pathway for cancer development. Our results show that PTPRG can reduce the phosphorylation of members of the Akt signaling pathway. In order to further confirm the role of Akt inhibition in tumor suppression in NPC cells, Akt inhibition in the two NPC tumorigenic PTPRG-down-regulated cell lines, HONE1 and HK1, was further investigated. A commercially available Akt-specific inhibitor, Akt XIII, was first utilized to inhibit the Akt activity in these two cell lines. After treating the HONE1 and HK1 cells with different concentrations of Akt inhibitor (DMSO control, 1.25 μM, 2.5 μM, 5 μM, and 10 μM), significant *in vitro* suppression of cell proliferation is observed (Figure 3A). The inhibitory effects increased with the increased concentrations of the Akt inhibitor (Figure 3A). Results suggested Akt inhibition plays a significant role in suppressing NPC cell growth.

In order to perform a more specific Akt inhibition experiment, Akt shRNA knockdowns were used for both HONE1 and HK1 cell lines to functionally evaluate the specific effect of inactivating the Akt signaling pathway in NPC cell lines. Akt knockdown experiments were performed using two sets of knockdown oligonucleotides (AKT984 and AKT1793) and the scramble oligonucleotides served as knockdown controls. Both sets of knockdown oligonucleotides showed a high efficiency for Akt knockdown in the two tested NPC cell lines (Figure 3B).

By using the panel of Akt knockdown oligonucleotides and the control, our results suggested that inhibition of Akt suppressed *in vitro* cell proliferation in both PTPRG-down-regulated HONE1 and HK1 cells (Figure 3C). The proliferation rate in HONE1 decreased from 100% with the scramble oligonucleotides (Ctrl) to 39.8% using the AKT984 knockdown oligonucleotide and 6.7% with the AKT1793 oligonucleotide. The same proliferation inhibition is also observed with the HK1 cell line; the proliferation rate decreased from 100% in the Ctrl to 71.4% with the AKT984 oligonucleotide and 67.8% with the AKT1793 oligonucleotide (Figure 3C). This demonstrates the inhibitory effect on cell proliferation by Akt inhibition in NPC.

In order to obtain functional evidence to support the suppressive role of PTPRG-associated Akt inhibition *in vivo*, the effects of Akt inhibition on the *in vivo* tumor growth were investigated using a nude mouse tumorigenicity assay. Knockdown of Akt in both HONE1 and HK1 cells induced significant *in vivo* tumor suppression, when compared to the control groups (*p* < 0.05) (Figure 3D). These results suggested that Akt knockdown reduces *in vivo* tumorigenicity and confirmed the important functional role of Akt inhibition on tumor suppression. The results also validated the role of PTPRG in tumor suppression [14] through inhibition of Akt signaling.

### PTPRG inhibits invasion and angiogenesis via Akt inhibition

Activation of invasion and angiogenesis are key hallmarks of cancer development [19]. As Akt signaling pathways can regulate invasion and angiogenesis processes, therefore, the regulatory roles of PTPRG in NPC invasion and angiogenesis were investigated.

**Figure 4:**
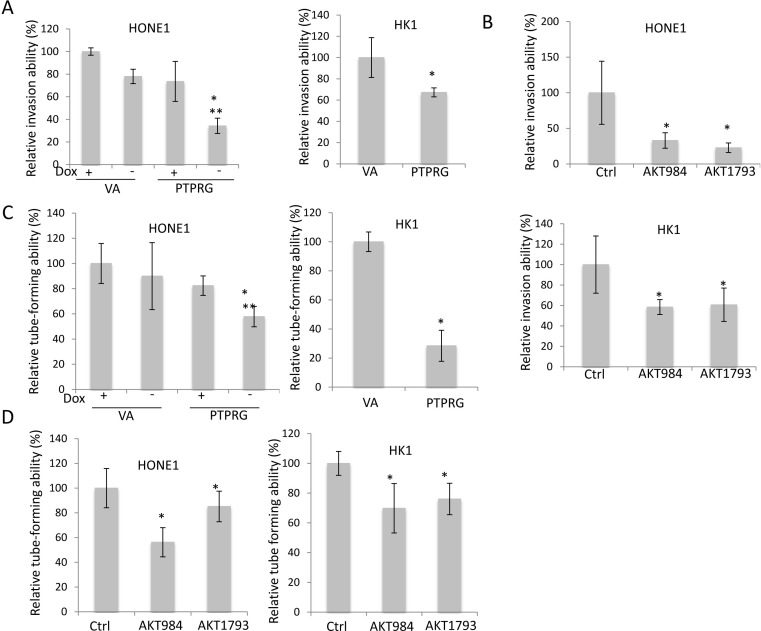
Re-expression of PTPRG and targeting Akt suppressed angiogenesis and invasion **A.** Re-expression of PTPRG in cancer cell lines, HONE1 and HK1, induced inhibition of invasion. For HONE1, the relative invasion ability was compared to the VA in the presence of Doxycycline (+Dox), and for HK1, the relative invasion ability was compared to the VA control. **B.** Akt knockdown inhibited the invasion ability of HONE1 and HK1. The relative invasion ability was compared to the scramble control (Ctrl). **C.** Re-expression of PTPRG reduced the tube-forming ability of HUVEC. For the CM from HONE1, the relative tube-forming ability was compared to the VA (+Dox), and for the CM from HK1, the relative invasion ability was compared to the VA control. **D.** Akt knockdown inhibited the tube-forming ability of HUVEC. The relative tube-forming ability was compared to the scramble control (Ctrl). * and **, *p* < 0.05, statistically significant differences compared with the VA and corresponding with dox control, respectively. Data are represented as mean + SD.

Results suggested that PTPRG was able to inhibit *in vitro* tumor invasion. Significant reduction of invasive abilities in the PTPRG-expressing HONE1 cells (34.3%) was observed, when compared to the VA control (+Dox; 100% and 78%, respectively), as well as when the PTPRG expression was switched off in the presence of doxycycline (+Dox; 73.6%) (Figure 4A). The invasion inhibitory effect was further confirmed in HK1; re-expression of PTPRG reduced invasion to 67.3%, when compared to the VA control (Figure 4A). The role of Akt inhibition on invasion suppression of cancer cells was also investigated; significant suppression of invasion was observed with both HONE1 and HK1 cell lines expressing both Akt knockdown oligonucleotides (32.9% and 22.74% for AKT984 and AKT1793 in HONE1, respectively, and 58.5% and 60.7% for AKT984 and AKT1793 in HK1, respectively) (Figure 4B). This suggested that targeting Akt via PTPRG could induce invasion suppression.

In addition to invasion suppression, Akt plays an important role in activating angiogenesis. The functional role for PTPRG regulation of angiogenesis was investigated by the HUVEC tube formation assay. Re-expression of PTPRG in both HONE1 and HK1 cell lines suppressed HUVEC tube-forming abilities (Figure 4C). Significant reduction of tube formation in the PTPRG-expressing HONE1 cells (57.8%) can be observed, when compared to the VA control (+Dox; 100% and 90.0%, respectively) and also when the PTPRG expression was switched off (+Dox; 82.4%). In HK1 cell lines, re-expression of PTPRG also inhibits tube formation. The HUVEC tube formation ability was reduced to 28.4% for PTPRG-expressing cells.

Targeting Akt in the PTPRG-down-regulated NPC cells also inhibited HUVEC tube formation (Figure 4D). Significant tube formation suppression was observed in both HONE1 and HK1 cell lines expressing both Akt knockdown oligonucleotides (69.8% and 76.1% for AKT984 and AKT1793 in HONE1, and 56.2% and 85.2% for AKT984 and AKT1793 in HK1, respectively). These results indicate that regulation of the Akt signaling by PTPRG is associated with anti-angiogenesis.

**Figure 5:**
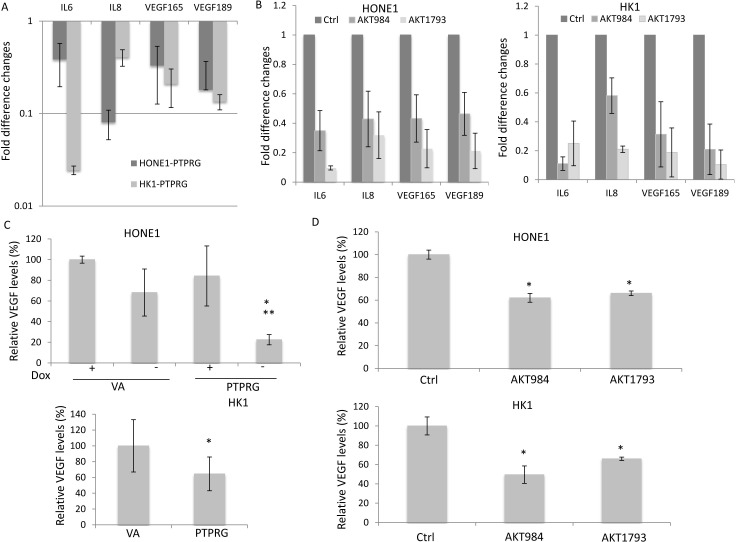
Re-expression of PTPRG and targeting Akt induced down-regulation of the Akt downstream post-angiogenic and invasion molecules **A.** QPCR results of PTPRG-expressing clones. Re-expression of PTPRG down-regulated the *IL6*, *IL8*, *VEGF165*, and *VEGF189* in both HONE1 and HK1. **B.** QPCR results of the HONE1 and HK1 after Akt knockdown. Knockdown of Akt down-regulated *IL6*, *IL8*, *VEGF165*, and *VEGF189* in both HONE1 and HK1. **C.** VEGF ELISA assay performed with CM of PTPRG-expressing clones. Re-expression of PTPRG reduced the secretion of VEGF protein in the CM. For HONE1 and HK1, the relative VEGF levels were compared to the VA (+Dox) controls. Data are represented as mean + SD. **D.** VEGF ELISA assay performed with CM of HONE1 and HK1 after Akt knockdown. Knockdown of Akt reduced the secretion of the VEGF in the CM. The relative VEGF levels were compared to the scramble control (Ctr). * and **, *p* < 0.05, statistically significant differences compared with the VA and corresponding with dox control, respectively. Data are represented as mean + SD.

PTPRG suppression of invasion and angiogenesis was further confirmed by measuring the expression of Akt downstream signaling molecules. The suppressive effects can contribute to down-regulation of Akt downstream pro-invasion and pro-angiogenesis factors, including IL6, IL8, and VEGF (165 and 189). These genes showed a trend of down-regulation in the PTPRG-expressing clones (Figure 5A) and in the Akt knockdown HONE1 and HK1 cells (Figure 5B). The protein secretion levels of the invasion- and angiogenesis-regulator, VEGF, in the conditioned media (CM) of the HONE1 and HK1 cells were confirmed by ELISA. Re-expression of PTPRG and Akt knockdown induced significant reduction of VEGF protein secretion in the CM (Figure 5C and 5D). Significant reduction of VEGF secretion in the PTPRG-expressing HONE1 cells to a level of 22.5% was observed, when compared to the VA control (+Dox), with levels of 100% and 68.2%, respectively, and also when the PTPRG expression was switched off (+Dox; 84.2%) (Figure 5C). Re-expression of PTPRG in the HK1 cell line reduced the secretion of VEGF to 64.5%. Targeting Akt also induced down-regulation of VEGF secretion in both HONE1 and HK1 cell lines. The secretion of VEGF was reduced to 69.8% and 76.1% for AKT984 and AKT1793 knockdowns in HONE1, respectively, and 56.2% and 85.2% for AKT984 and AKT1793 in HK1, respectively. This is consistent with the functional assay results showing PTPRG inhibits invasion and angiogenesis of the cancer cells and associates with the regulation of the Akt signaling pathway.

### Knockdown of Akt in NPC cells suppresses *in vivo* invasion

**Figure 6:**
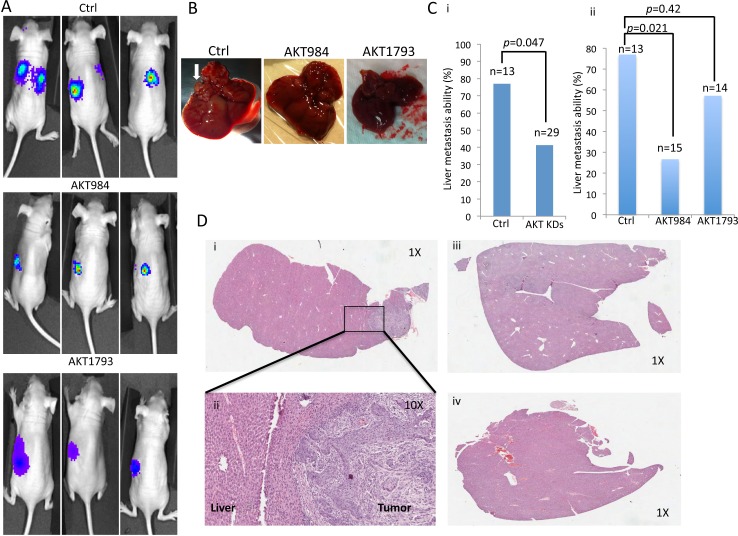
*In vivo* metastasis assay of cancer cells after Akt knockdown in mice by intrasplenic injection **A.** Representative images of the *in vivo* animal imaging system of the scramble control (Ctrl), and AKT984 and AKT1793 knockdown constructs. Representative images for the metastasis are observed with the scramble control group. The bioluminescence signals indicated the location of the cancer cells. **B.** Mice after the bioluminescence signal detection were sacrificed and dissected. The livers were excised and the presence of metastasis was investigated by histology. Lesions in the livers were observed in the liver if metastasis occurred. Representative images of lesions in the livers are indicated by a white arrow. **C.** Summary of the liver metastasis ability of the cancer cells. High percentage of metastasis of the cancer cells was observed in the scramble control (Ctrl), while the Akt knockdown constructs (AKT984 and AKT1793) showed a relatively low trend of metastasis. Comparison of the metastatic rate of the Ctrl to a (i) combination of the two knockdown groups (*p =* 0.047) and (ii) two individual knockdown groups. **D.** Histological analysis of the livers from the experimental mice to confirm the existence of tumor cells in the mouse livers. Representative H&E staining images of liver tissues from the Ctrl and Akt knockdown constructs. (i and ii) Livers from the scramble group (Ctrl), which were identified in mice with metastases using different magnifications (1X and 10X). (iii and iv) Livers from the AKT knockdown groups (AKT984 and AKT1793) were identified in mice with no observable metastasis.

To further evaluate the contribution of targeting the Akt signaling in *in vivo* metastasis, a luciferase-labelled metastatic NPC cell line, HONE1, was used for intrasplenic injection in nude mice. The *in vivo* metastasis of the scramble control and the Akt knockdown clones was monitored by the Xenogen live imaging system (Figure 6A). The HONE1 cell line showed a high metastasis rate to the liver region. Visible tumor nodules (Figure 6B) were observed in 77% of livers in the control group. A statistically significant metastatic inhibition (*p =* 0.047) was observed for the AKT knockdown groups (41%) versus the Ctrl group (Figure 6Ci). A trend of decreased metastasis to 27% and 57% was observed in the two Akt knockdown clones, AKT984 and AKT1793, respectively, when compared to the Ctrl group (Figure 6Cii). The results were further validated by H&E staining (Figure 6D); representative H&E staining images of liver tumor nodules (Figure 6Di and 6Dii) and metastasis-negative samples (Figure 6Diii and 6Div) are shown. These results provide evidence for the functional role of Akt inhibition in suppression of *in vivo* metastasis.

## DISCUSSION

Clarification of the molecular mechanisms for NPC development is still unclear and oncogenic changes in different signaling pathways occurring during malignant transformation need further study. In this current study we demonstrate the functional role of a previously identified gene of interest in NPC, *PTPRG,* which belongs to the PTP family. Ongoing research of PTP family members suggests their importance in modulating cellular signaling pathways, which are associated with cancer development. A recent head and neck cancer study confirmed that mutation of PTPRT results in increased phosphor-STAT3 levels in the head and neck squamous cell carcinoma tumors [20]. Our gene of interest, *PTPRG*, was previously identified as an important tumor suppressor in NPC. However, its function in NPC was still unclear; since little is known about its protein interacting partners and role in regulating cellular signaling pathways. Here, we provide evidence to support the regulatory role of this PTP in orchestrating the deregulated oncogenic Akt signaling pathway in NPC cancer cells.

Our results confirm the interaction of PTPRG and EGFR, and the ability of PTPRG to reduce EGFR phosphorylation in NPC. Interestingly, besides PTPRG, other PTP family members, PTPRS [21] and PTP1B [22], also play critical roles in dephosphorylation and inactivation of EGFR. EGFR is a member of the RTK family and it is one of the important cancer-related signaling regulators. EGFR contains several phosphorylation sites. After dimerization and binding of ligands, including the EGF and TGF-α [23], some phosphorylation sites initiate self-phosphorylation and activate EGFR function [24]. These phosphorylated sites then serve as a docking bay for recruitment and activation of downstream signaling molecules. Deregulation of the EGFR phosphorylation contributes to cancer. Previous study suggested that inhibition of EGFR-Y1068 and -Y1086 auto-phosphorylation resulted in attenuation of the cancer cell transformed phenotype [25]. EGFR-Y1068 was shown to be an important binding site for a growth receptor binding protein Grb2 [26] and scaffold protein Gab1 to activate PI3K/Akt [27] and MAPK [28] signaling molecules. The activation of PI3K/Akt is negatively regulated by a well-studied tumor suppressor PTEN in the PTPase family and results in tumor suppression [7]. Interestingly, a previous study of PTPRG in breast cancer also confirmed that PTPRG regulates the ERK1/2 pathway [16], which is also one of the key EGFR-regulated signaling pathways. Furthermore, a recent study in leukocytes also validated the role of PTPRG in dephosphorylation of EGFR-Y1068 [30]. These results further illustrate the important functional role of PTPRG in regulating the EGFR phosphorylation and, thus, modulating its downstream Akt and ERK signaling pathways.

Clinically, up-regulation of EGFR can be detected in NPC patient biopsies [31], as PTPRG can induce dephosphorylation of EGFR and, thus, the downstream PI3K/Akt signaling pathway; this further supports the critical tumor suppressive role of PTPRG in NPC. It is well-known that Akt is a central signaling molecule modulating many cellular processes and its deregulation is crucial for activation of tumorigenic characteristics including uncontrolled cell proliferation, angiogenesis, invasion, and metastasis. Previous studies indicated deregulation of Akt signaling in NPC [1]. Akt helps to maintain the cyclin D1 levels via activation of transcription factor Ap1 (c-jun/c-fos) [32] and inhibition of ubiquitin-mediated cyclin D1 degradation [22, 33]. This is consistent with our previous finding that *PTPRG* re-expression induced cell cycle G_0_/G_1_ arrest in NPC [14] via down-regulation of cyclin D1 protein and, thus, reduced pRB phosphorylation [14]. The effects of Akt inhibition were further validated by Akt knockdown in the PTPRG-down-regulated NPC cell lines [14]. These experiments provide direct evidence to support the functional effects of Akt inactivation in cancer and support our finding showing that PTPRG re-expression inhibits cell proliferation and *in vivo* tumor growth via Akt inhibition.

PTPRG also plays an anti-angiogenesis role in NPC through regulating the expression of EGFR/Akt signaling downstream molecules, VEGF, IL6, and IL8. VEGF is one of the key pro-angiogenic proteins. In NPC, the oncogenic role of VEGF was confirmed; elevated VEGF expression is associated with poor overall survival in NPC patients [34]. Inhibition of the Akt by PI3K siRNA knockdowns induced VEGF protein down-regulation [35, 36] and inhibition of angiogenesis. Apart from VEGF, Akt also controlled the expression of the pro-angiogenesis proteins IL6 and IL8 [37], which are also are involved in regulation of angiogenesis in NPC [38]. Re-expression of PTPRG suppresses EGFR/Akt signaling activities and induces down-regulation of the pro-angiogenic proteins, VEGF, IL6, and IL8. This further supports the important function of PTPRG in regulating a hallmark of cancer, namely angiogenesis, in NPC through Akt signaling inhibition.

In addition to promotion of angiogenesis, pro-angiogenic proteins, VEGF, IL6, and IL8, can also initiate tumor invasion and metastasis. Intense staining of VEGF protein is observed in the advanced squamous cell carcinoma tissues [39] and is positively correlated with invasion and metastasis [40]. High expression of VEGF in pancreatic adenocarcinoma samples associates with liver metastasis [41]. Increasing evidence supports the involvement of both IL6 and IL8 in inducing metastasis in cancer. IL6 was confirmed to promote head and neck tumor metastasis [42]. The serum IL6 and IL8 levels were significantly increased in the colorectal cancer patients with liver metastasis [43]. A previous study also suggests that suppression of the PI3K/Akt signaling results in reducing metastasis in NPC [44]. These findings further validate the role of PTPRG in suppressing tumor invasion and metastasis via inhibition of Akt signaling and downstream VEGF, IL6, and IL8 and, thus, inhibiting tumor invasion and liver metastasis in NPC, as identified in this current study.

Several lines of evidence emphasize that inhibition of the Akt signaling suppresses the cell proliferation, angiogenesis, invasion, and most importantly, metastasis. We confirm the vital role of *PTPRG* in dephosphorylation and inactivation of EGFR/Akt signaling and, in turn, the downstream Akt signaling pathway cascade to promote oncogenesis and NPC development.

## MATERIALS AND METHODS

### Cell culture

NPC cell lines, HONE1 and HK1, were used in this current study. The tetracycline transactivator tTA-producing *PTPRG*-expressing HONE1 clone and vector-alone (VA) were cultured as previously described [14, 17]. The HK1 was cultured as previously described [45]. The metastatic HONE1 cell line was labeled with luciferase markers as described [46] for the *in vivo* imaging to monitor metastasis. All cell lines used in this current study were obtained from the Hong Kong NPC AoE Cell Line Repository and have been authenticated using the AmpFlSTR Identifier PCR Amplification kit (Life Technologies).

### Phosphorylation antibody array

A human phosphokinase antibody array (R&D Systems) was utilized in this current study. The cell culture, protein preparation, hybridization, and signal detection were performed as described by the manufacturer. The intensity of the signals was quantified by the BioRad Quantity ONE software using the Molecular Imager Doc XR System (BioRad Laboratories) as previously described [47]. The vector-alone (VA) and PTPRG-expressing clones were used for the phosphorylation array analysis.

### Co-immunoprecipitation (CoIP)

The CoIP experiment was performed as previously described [38]. In brief, cells were seeded on 150 mm culture plates for 48 hours to obtain sufficient input material for the CoIP reaction. The non-denaturing lysis buffer was used for protein extraction. The cell lysate was then immunoprecipitated with antibodies specific to EGFR and PTPRG (Supplementary Table 2) and IgG served as a control

### Western blot analysis

For the molecular signaling study, the cells were seeded onto the cell culture plate to obtain cell lysates for the WB analysis. For the inducible clones, the doxycycline was removed 16 hours after cell seeding and the cells then cultured in the media without doxycycline for 24 hours. For the other cell lines, the cells were seeded onto culture plates for 24 hours for cell lysate collection. The cell lysate was collected and WB analysis was performed as previously described [14]. The information on antibodies used in this current study is summarized in Supplementary Table 2.

### AKT1 knockdown construct and AKT inhibitor

The pLKO TRC cloning vector (Addgene no. 10878) mammalian expression lentiviral plasmid was used for the Akt knockdown experiment. The knockdown constructs were designed according to the instructions of the Public TRC portal. Two sets of knockdown oligonucleotides, which targeted Akt, Akt984 (5′-CCGGCGCGTGACCATGAACGAGTTTCTGCAG AAACTCGTTCATGGTCACGCGTTTTTG-3′) and Akt1793 (5′-CCGGGCAGCACGTGTACGAGAAGAACTGCAG TTCTTCTCGTACACGTGCTGCTTTTTG-3′), were used for the knockdown experiment. The scramble vector (Addgene 1864) served as a control. The AKT inhibitor XIII (Calbiochem) was used in this study. Cells were treated with the inhibitor for 48 hours and then the cell proliferation rate was investigated.

### *In vivo* nude mouse tumorigenicity and metastasis assays

All *in vivo* study protocols in this current study were approved by the Committee on the Use of Live Animals in Teaching and Research, The University of Hong Kong and the Department of Health, Hong Kong SAR. All mice were kept in the Laboratory Animal Unit of the University of Hong Kong according to the Associating for Assessment and Accreditation of Laboratory Animal Care (AAALAC) international guidelines. The *in vivo* nude mouse tumorigenicity assay was conducted as previously described [14]. In brief, a total of 1 × 10^7^ of HONE1 and of HK1 cells were injected into athymic BALB/c Nu/Nu six to eight-week old nude mice subcutaneously. The tumor sizes were measured weekly. For the *in vivo* metastasis assay, intrasplenic injection in nude mice was utilized. A total of 1 × 10^6^ of cells was injected into the spleen of the mouse. Metastasis of different tested experimental groups was monitored by intraperitoneal injection of D-Luciferin (150 mg/kg; StayBrite, Biovision) into the mouse. The bioluminescent signal was detected using the Xenogen IVIS 100 *in vivo* Imaging System in the Faculty Core Facility, University of Hong Kong. After three weeks inoculation, the livers of the mice were excised and examined by a pathologist to assess liver metastasis frequency (no. mice showing liver metastasis/no. mice in study group X 100%). The mouse livers were then paraffin embedded for further histological analysis.

### HUVEC tube formation assay

The CM was prepared by adding serum-free media to the cells and after 16 hours of incubation, the CM for HUVEC tube formation assay was collected and the assay performed as previously described [38, 48]. The total tube length from at least three different fields was measured using SPOT software (Diagnostic Instrument).

### Real-time quantitative RT-PCR

Q-PCR was performed as previously described [14]. The *IL6, IL8, VEGF165,* and *VEGF189* primers were designed as described [38, 47, 49].

### Real-time cell proliferation and invasion assays

The real-time cell proliferation and invasion assays were conducted using the E-plate and the CIM-plate, respectively, with the xCelligence system (Roche) according to the manufacturer's instructions and as previously described [50, 51].

### VEGF ELISA analysis

The CM was prepared as described in previous section. CM was collected for investigation of the VEGF protein levels. The VEGF protein levels in the tested and control groups were measured by utilizing the Quantikine Human VEGF immunoassay system (R&D Systems) according to the manufacturer's instructions as previously described [38, 48]. The absorbance was measured by the Labsystem Multiskan MS Plate Reader (Thermo Fisher Scientific).

### Paraffin embedding and Hematoxylin and Eosin (H&E) staining

The paraffin embedding and H&E staining were performed as previously described [14].

### Statistical analysis

The results of *in vitro* assay represent the arithmetic mean + SD of determinations. Student's *t* test was used to determine the confidence levels in group comparisons. The SEM was used to calculate the SE of the *in vivo* assay. The Fisher's exact test was used to determine the confidence levels of the *in vivo* metastasis. A *p-*value <0.05 was considered statistically significant.

## SUPPLEMENTARY MATERIAL AND TABLES


