# Supplementary material for: PTPRG suppresses tumor growth and invasion via inhibition of Akt signaling in nasopharyngeal carcinoma
Source: Oncotarget. 2015 Apr 19;6(15):13434–47. doi: 10.18632/oncotarget.3876 (PMC4537025; doi:10.18632/oncotarget.3876)
Supplement: Supplementary file 1 [file oncotarget-06-13434-s001.pdf]

PTPRG suppresses tumor growth and invasion via inhibition of Akt signaling in nasopharyngeal carcinoma

Supplementary Material

Supplementary Table 1: The detailed information and map of the phosphorylation array

	1	2	3	4	5	6	7	8	9	10	11	12	13	14	15	16	17	18
A	Ref spot	Ref spot	p38α-T180/Y182	p38α-T180/Y182	ERK1/2-T202/Y204, T185/Y187	ERK1/2-T202/Y204, T185/Y187	JNK pan-T183/Y185, T221/Y223	JNK pan-T183/Y185, T221/Y223	GSK-3α/β-S21/S9	GSK-3α/β-S21/S9			p53-S392	p53-S392			Ref spot	Ref spot
B			MEK1/2-S218/S222, S222/S226	MEK1/2-S218/S222, S222/S226	MSK1/2-S376/S360	MSK1/2-S376/S360	AMPKα1-T174	AMPKα1-T174	Akt-S473	Akt-S473	Akt-T308	Akt-T308	p53-S46	p53-S46				
C	TOR-S2448	TOR-S2448	CREB-S133	CREB-S133	HSP27-S78/S82	HSP27-S78/S82	AMPKα2-T172	AMPKα2-T172	β-catenin	β-catenin	p70S6 kinase-T389	p70S6 kinase-T389	p53-S15	p53-S15	p27-T198	p27-T198	Paxillin-Y118	Paxillin-Y118
D	Src-Y419	Src-Y419	Lyn-Y397	Lyn-Y397	Lck-Y394	Lck-Y394	STAT2-Y689	STAT2-Y689	STAT5a-Y694	STAT5a-Y694	p70S6 kinase-T421/S424	p70S6 kinase-T421/S424	RSK1/2/3-S380, S386, S377	RSK1/2/3-S380, S386, S377	p27-T157	p27-T157	PLCγ-1-Y783	PLCγ-1-Y783
E	Fyn-Y420	Fyn-Y420	Yes-Y426	Yes-Y426	Fgr-Y412	Fgr-Y412	STAT3-Y705	STAT3-Y705	STAT5b-Y699	STAT5b-Y699	p70S6 kinase-T229	p70S6 kinase-T229	RSK1/2-S221/S227	RSK1/2-S221/S227	c-jun-S63	c-jun-S63	Pyk2-Y402	Pyk2-Y402
F	Hck-Y411	Hck-Y411	Chk-2-T68	Chk-2-T68	FAK-Y397	FAK-Y397	STAT6-Y641	STAT6-Y641	STAT51/b-Y694/Y699	STAT51/b-Y694/Y699	STAT1-Y701	STAT1-Y701	STAT4-S693	STAT4-S693	eNOS-S1177	eNOS-S1177	PBS (-ve control)	PBS (-ve control)
G	Ref spot	Ref spot			PBS (-ve control)	PBS (-ve control)												

Supplementary Table 2: List of antibodies used in this current study

Name	HOST	Phosphorylated site	Company	Catalog no.
EGFR	Rabbit	N/A	Cell Signaling	4267
p-EGFR-Y1068	Rabbit	Tyr1068	GeneTex	GTX61057
p-EGFR-Y1068	Rabbit	Tyr1086	GeneTex	GTX61058
Gab1	Rabbit	N/A	Cell Signaling	3232
p-Gab1-Y307	Rabbit	Tyr307	Cell Signaling	3234
p-Gab1-Y627	Rabbit	Tyr627	Cell Signaling	3233
PI3K-p85	Rabbit	N/A	Cell Signaling	4228
p-PI3K-p85-Y458	Rabbit	Tyr458	Cell Signaling	4257
PDK1	Rabbit	N/A	Cell Signaling	3062
p-PDK1-S241	Rabbit	Ser241	Cell Signaling	3438
Akt	Rabbit	N/A	Cell Signaling	9272
pAkt-S473	Rabbit	Ser473	Cell Signaling	4058
p-Akt-T308	Rabbit	Thr308	Cell Signaling	2965
JNK	Rabbit	N/A	Cell Signaling	9252
p-JNK-T183/Y185	Mouse	Thr183/Tyr185	Cell Signaling	9255
c-jun	Rabbit	N/A	Cell Signaling	9165
p-c-jun-S63	Rabbit	Ser63	Cell Signaling	2361
p38α	Rabbit	N/A	Cell Signaling	9212
p-p38α-T180/Y182	Mouse	Thr180/Tyr182	Cell Signaling	9216
CREB	Goat	N/A	R&D	AF2989
p-CREB-S133	Rabbit	Ser133	R&D	AF2510
PTPRG	Rabbit	N/A	Abcam	ab37525
